# Structural Insights into the Quaternary Catalytic Mechanism of Hexameric Human Quinolinate Phosphoribosyltransferase, a Key Enzyme in *de novo* NAD Biosynthesis

**DOI:** 10.1038/srep19681

**Published:** 2016-01-25

**Authors:** Hyung-Seop Youn, Tae Gyun Kim, Mun-Kyoung Kim, Gil Bu Kang, Jung Youn Kang, Jung-Gyu Lee, Jun Yop An, Kyoung Ryoung Park, Youngjin Lee, Young Jun Im, Jun Hyuck Lee, Soo Hyun Eom

**Affiliations:** 1 School of Life Sciences, Gwangju Institute of Science and Technology, Gwangju 500-712, South Korea; 2 Steitz Center for Structural Biology, Gwangju Institute of Science and Technology, Gwangju 500-712, South Korea; 3 College of Pharmacy, Chonnam National University, Gwangju 500-757, South Korea; 4 Division of Polar Life Sciences, Korea Polar Research Institute, Incheon 406-840, South Korea; 5 Department of Polar Sciences, Korea University of Science and Technology, Incheon 406-840, South Korea; 6 Department of Chemistry, Gwangju Institute of Science and Technology, Gwangju 500-712, South Korea

## Abstract

Quinolinate phosphoribosyltransferase (QPRT) catalyses the production of nicotinic acid mononucleotide, a precursor of *de novo* biosynthesis of the ubiquitous coenzyme nicotinamide adenine dinucleotide. QPRT is also essential for maintaining the homeostasis of quinolinic acid in the brain, a possible neurotoxin causing various neurodegenerative diseases. Although QPRT has been extensively analysed, the molecular basis of the reaction catalysed by human QPRT remains unclear. Here, we present the crystal structures of hexameric human QPRT in the apo form and its complexes with reactant or product. We found that the interaction between dimeric subunits was dramatically altered during the reaction process by conformational changes of two flexible loops in the active site at the dimer-dimer interface. In addition, the N-terminal short helix α1 was identified as a critical hexamer stabilizer. The structural features, size distribution, heat aggregation and ITC studies of the full-length enzyme and the enzyme lacking helix α1 strongly suggest that human QPRT acts as a hexamer for cooperative reactant binding via three dimeric subunits and maintaining stability. Based on our comparison of human QPRT structures in the apo and complex forms, we propose a drug design strategy targeting malignant glioma.

Nicotinamide adenine dinucleotide (NAD), a ubiquitous coenzyme, exists in both oxidised (NAD^+^, electron acceptor) and reduced (NADH, electron donor) forms. NAD is essential for cell survival and plays a key role in oxidative phosphorylation in the cellular respiratory chain. In addition to redox reactions such as oxidative phosphorylation, NAD^+^ is involved in various cellular processes including genomic repair and stability, chromatin modulation, calcium homeostasis, aging and apoptosis^1^,^2^,^3^,^4^,^5^. In particular, most cancer cells produce NAD^+^ to sustain rapid growth and to tolerate DNA damage and genetic instabilities caused by alkylating agent treatment by acting as a substrate of poly (ADP-ribose) polymerase^5^,^6^,^7^. Quinolinate phosphoribosyltransferase (QPRT, EC 2.4.2.19) belongs to the phosphoribosyltransferase (PRT) family and is involved in *de novo* NAD biosynthesis using quinolinic acid (QA) as a precursor in both prokaryotes and eukaryotes^8^. QPRT catalyses the transfer of QA converted from tryptophan and 5-phosphoribosyl-1-pyrophosphate (PRPP), yielding carbon dioxide, pyrophosphate and nicotinic acid mononucleotide (NAMN), which is a precursor of nicotinate adenine dinucleotide followed by the conversion to NAD (Supplementary Fig. S1). QA is the first intermediate in the *de novo* synthesis of NAD, which is produced via the degradation of tryptophan through the metabolic cascade in the brain known as the kynurenine pathway^9^. In mammals, QA is a potent excitotoxic compound in the central nervous system that causes neuronal damage via continuous activation of *N*-methyl-D-aspartate receptors^10^. QPRT plays a prominent role in QA homeostasis in the brain, therefore, malfunction of QPRT increases QA levels, which is strongly involved in a series of severe neurodegenerative disorders including Huntington’s disease, Alzheimer’s disease, Epilepsia and AIDS dementia complex^11^,^12^,^13^,^14^,^15^,^16^,^17^,^18^. Regulation of the QA level by QPRT *in vivo* affects the kynurenine metabolic pathway in the brain, liver and kidney, and contributes to the neuropathological conditions and immune-activated diseases^18^. QPRT also inhibits the apoptotic inducer active caspase-3, suppressing the spontaneous cell death pathway^19^. Recently, QPRT has attracted attention as a target protein essential for the survival of malignant glioma, a type of tumour consisting of glial cells in the central nervous system^20^,^21^.

QPRT structures from various organisms have been previously reported. All structures exhibit typical features of the type II PRT structure including an N-terminal four-stranded open-face β-sandwich domain and a C-terminal α/β barrel domain, unlike the α_4_/β_5_ folds of other members of PRT. QPRTs have been shown to exist as dimers or hexamers^22^,^23^,^24^,^25^,^26^,^27^,^28^,^29^. QPRTs from eukaryotes, including human (*Hs*QPRT), porcine (*Ss*QPRT), rat and yeast (*Sc*QPRT), are known to be hexamers in solution^22^,^30^,^31^,^32^, which is consistent with the results of structural studies of *Hs*QPRT in complex with a tartrate, which mimics part of QA and *Sc*QPRT, demonstrating that these molecules form hexameric structures. However, most prokaryotic QPRTs exist as dimers, although the enzymes from *Helicobacter pylori* and *Thermus thermophilus* have a hexameric structure that is identical to *Hs*QPRT-tartrate and *Sc*QPRT^23^,^24^,^26^,^27^. Based on its mechanical kinetics, the F181P mutant in the dimer-dimer interface of QPRT from *Helicobacter pylori* (*Hp*QPRT) changed the oligomer from a hexamer to a dimer, which exhibited dramatically reduced enzyme stability and non-functionality^33^. Although numerous structures of *Hs*QPRT have been determined in one apo form and two complexes with reactant QA analogues (tartrate and phthalate), the molecular basis for the existence of *Hs*QPRT and the catalysis of the phosphoribosyl transfer reaction as a hexamer remains unclear. Here, we report the crystal structures of *Hs*QPRT in the apo form with different conformations and the QA (*Hs*QPRT-QA)- and NAMN (*Hs*QPRT-NAMN)-bound forms. Comparison of the apo enzyme structures reveal that, the reactant (QA) and the reaction product (NAMN) complexes have a conformational change that dramatically affected two distinctive flexible loops around the active site as well as the interface forming the hexamer, indicating that the reaction mediated by hexameric *Hs*QPRT is regulated by the interaction between two dimeric subunits. We also found that the N-terminal helix of *Hs*QPRT was critical for hexamer formation. Our results provide structural insight into the concerted mode of the reactant binding and hexamer stabilisation of *Hs*QPRT. In addition, analysis of the range of structural diversity of QPRTs has clinical implications in the development of anticancer agents in the treatment of malignant glioma.

## Results

### Overall structure of human QPRT

**Figure 1:**
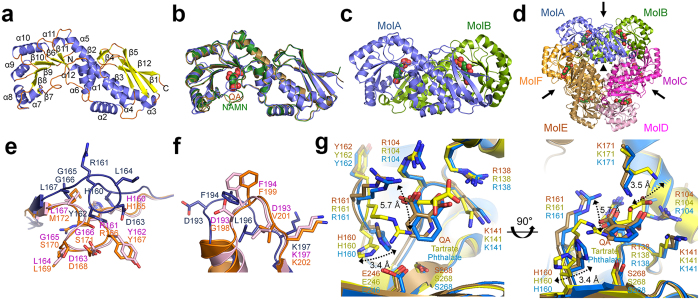
Overall structure and comparison of *Hs*QPRT. (**a**) Monomeric structure of *Hs*QPRT. The 12 α-helices and 12 β-sheets represent α1–α12 and β1–β12, respectively. (**b**) Superimposition of the overall structures of monomeric *Hs*QPRT in the apo form (blue), *Hs*QPRT-QA (brown) and *Hs*QPRT-NAMN (green). (**c**) Dimeric structure of *Hs*QPRT. Substrate molecules (NAMN) are shown as spheres. (**d**) Hexameric structure of *Hs*QPRT indicated a trimer of dimers (MolA-MolB, MolC-MolD and MolE-MolF). The two- and three-fold axes of the hexamer are indicated as arrows and triangles, respectively. (**e,f**) Structural comparison among *Hs*QPRTs in *Hs*QPRT-open (blue), *Hs*QPRT-closed (magenta) and yeast apo enzyme (orange) in loop L (**e**) and loop O (**f**). (**g**) Structural comparison of *Hs*QPRT in complex with QA (brown), tartrate (yellow) and phthalate (blue). Conformational changes are shown as two-sided arrows.

To investigate the molecular basis of mammalian QPRT in the hexamer state upon substrate binding, we determined the structures of *Hs*QPRT in the apo form and in complex with its reactant QA as well as the reaction product NAMN at 2.8, 3.1 and 2.6 Å, respectively. The data collection and refinement statistics are summarised in Table 1. The *Hs*QPRT monomer consists of 12 α-helices and 12 β-strands arranged into two structural domains: an N-terminal open-faced α/β-sandwich domain (residues 1–112 and 279–288; six-stranded antiparallel β-sheets and five α-helices) and a C-terminal open TIM α/β-barrel domain (residues 113–278; six β-strands and seven α-helices) (Fig. 1a). The domain architecture and secondary structure elements were nearly the same in both the apo form and the substrate complexes (Fig. 1b). The dimer of *Hs*QPRT is formed by a head-to-tail interaction of the N-terminal domain of one monomer and the C-terminal domain of the other monomer (Fig. 1c). The active sites of the twisted bow tie-shaped *Hs*QPRT dimer are located at the interface between the α/β-barrel of one subunit and the β-sandwich of the other subunit. The active site consists of the C-terminal α/β-barrel with one N-terminal β-sheet for dimerisation, which is consistent with the dimeric interfaces found in other type II PRTs^27^,^34^. Each monomer extensively connects to three other monomers, indicating that *Hs*QPRT forms intra- and inter-contacting arrangements in the hexameric assembly.

Hexameric *Hs*QPRT is formed by the trimerisation of dimers in a three-fold axis, which is similar to other eukaryotic enzymes such as *Ss*QPRT and *Sc*QPRT (Fig. 1d and Supplementary Fig. S2). Size exclusion chromatographic analysis revealed that *Hs*QPRT forms a hexamer with an estimated molecular weight of approximately 190 kDa. Based on biochemical approaches, this hexamerisation is consistent with QPRTs from other mammals such as rat and porcine^28^,^29^,^30^,^31^.

### Structural comparison of human and other eukaryotic QPRTs

Various structures of eukaryotic QPRTs have been reported, such as those from human (apo and complexes with tartrate or phthalate, an analogue of QA with a benzene ring rather than a pyridine ring)^26^,^29^, porcine (NAMN complex)^28^ and yeast (apo, complexes with QA, PRPP or phthalate)^27^. The apo *Hs*QPRT structure was compared with previously reported *Hs*QPRT and *Sc*QPRT apo structures (PDB codes 4KWV and 3C2E)^27^,^29^. However, the apo structure of *Hs*QPRT described by Malik *et al.* is relatively similar to that of *Sc*QPRT. Moreover, our apo *Hs*QPRT structure showed a different conformation from other structures, particularly in loop L (H160–L167) and loop O (A191–V198) (Fig. 1e,f). Two loops were located in not only the active site but also the dimer-dimer interface. The orientations of these loops in our apo structure resembled an open structure, whereas that in the other apo structure appeared as a more closed dimer-dimer interface, referred to as *Hs*QPRT-open and *Hs*QPRT-closed, respectively. Conformational changes in loops L and O suggest that apo *Hs*QPRT exists as a dynamically equilibrated structure around six active sites located in three dimer-dimer interfaces, potentially affecting substrate binding and enzyme turnover rate.

In addition to analysing complex structures, we also compared the structures of QPRT in complex with QA (reactant of *Hs*QPRT), tartrate (PDB code 2JBM) and phthalate (PDB code 4KWW) to investigate the differences in the binding mode between the substrate and its mimic. Both loops L and O in the two structures of *Hs*QPRT in complex with QA analogues were more similar to *Hs*QPRT-closed than to *Hs*QPRT-open. Tartrate has a hydroxyl ethyl group rather than the pyridine ring of QA, which induces conformational changes in H160 and R161 (Fig. 1g). Although R161 showed an ionic interaction with a carboxylate in both QA and tartrate, R161 of the tartrate complex protruded into the pyridine moiety of QA and created an additional interaction with E246 and S268. Alternatively, R161 of *Hs*QPRT-QA rotated by approximately 50° to avoid the steric hindrance against the pyridine moiety and resulted in a conformational change of the imidazole side chain of H160 in addition to its interaction with the carboxylate moiety of QA. Moreover, the ε-amino group of K171 was rotated by approximately 45° because of the difference in the position of the carboxylate moiety of QA and tartrate molecules of 2.4 Å. Phthalate showed a highly similar mode of binding to that of QA, making *Hs*QPRT the stable intermediate state that could not combine with PRPP to produce NAMN.

In addition to in humans, the NAMN-bound QPRT structure from porcine (*Ss*QPRT-NAMN, PDB code 4I9A) showed structural and sequence conservation, particularly near the active site. Although the NAMN binding site is similar between human and porcine, residues near the dimer-dimer interface showed different conformations (Supplementary Fig. S3). In MolA, the residue corresponding to R190 of *Ss*QPRT-NAMN is glutamine in human and was rotated by approximately 180°. In addition, the human protein contains D193 rather than N193 from porcine, enabling higher ionic contacts with H133′ in MolF and indicating that *Hs*QPRT forms a more stable hexameric configuration compared to the porcine enzyme.

### Changes in the dimer-dimer interaction upon substrate binding

**Figure 2:**
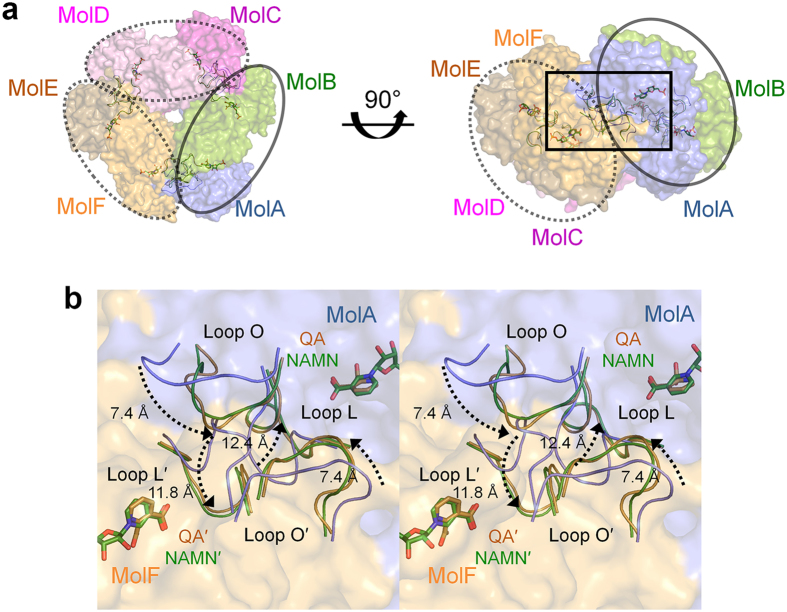
Conformational changes in loops L and O upon substrate binding. (**a**) Conformational changes in hexameric *Hs*QPRT-open (blue), *Hs*QPRT-QA (brown) and *Hs*QPRT-NAMN (green). Dimeric subunits are represented as straight or dashed circles. (**b**) Detailed view. Loops of adjacent dimer (MolF) are labelled as primed. Conformational changes in loops L and O from *Hs*QPRT-open to substrate complexes are displayed as dashed arrows (stereographic views).

**Figure 3:**
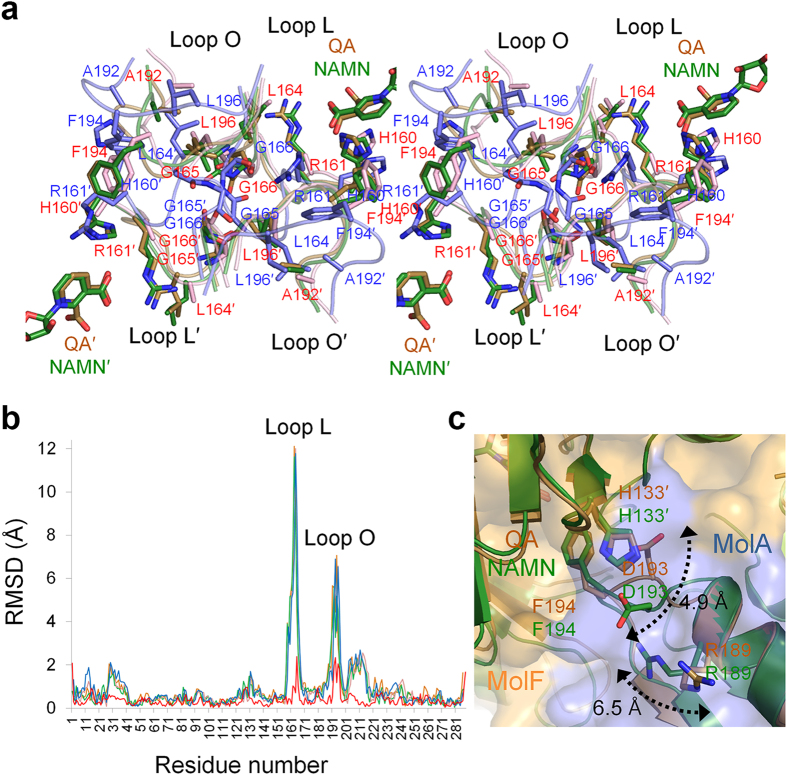
Detailed view of structural comparison of *Hs*QPRTs. (**a**) Detailed structural comparison of loops L and O of *Hs*QPRT upon substrate binding of *Hs*QPRT-open (blue), *Hs*QPRT-QA (brown), *Hs*QPRT-NAMN (green) and *Hs*QPRT-closed (magenta). Residues of *Hs*QPRT-open and the other structures having similar conformations (*Hs*QPRT-QA, *Hs*QPRT-NAMN and *Hs*QPRT-closed) are labelled blue and red, respectively. (stereographic views). (**b**) Root mean square deviations (RMSDs) of *Hs*QPRT-QA (brown), *Hs*QPRT-NAMN (green), *Hs*QPRT-closed (magenta) and phthalate complex (blue) based on *Hs*QPRT-open as a reference. RMSD between *Hs*QPRT-QA and *Hs*QPRT-NAMN is coloured in red. (**c**) Structural comparison of *Hs*QPRT-QA (brown) and *Hs*QPRT-NAMN (green). Residues showing different conformations are indicated as two-sided arrows.

Although the overall folds of hexameric *Hs*QPRT-open and substrate complexes were highly similar, specific regions of the structures differed, particularly the dimer-dimer interfaces (Fig. 2a,b). In the MolA of the *Hs*QPRT-open structure, loop L and loop O located in the dimer-dimer interface formed extensive hydrophobic interactions with loop O and loop L in the subunit of the adjacent dimer (MolF). Two flexible loops showed major differences upon substrate binding, which was not introduced in all QPRT structures determined previously including apo structures (Fig. 3a). Both loops L and O were nearly the same in the two complex structures (QA and NAMN) as well as in *Hs*QPRT-closed (Fig. 3a,b). In the *Hs*QPRT-open structure, loop L of MolA had a compact interaction with MolF. In particular, L164 in loop L showed a hydrophobic interaction with A192′, F194′ and L196′ in loop O of the adjacent subunit. However, the ionic interactions of H160 and R161 with the carboxyl moiety of both QA and NAMN induced overall rearrangement of loop L and a break in the contact between the two dimers that constituted the interaction between loop L of MolA and loop O′ of MolF. In the structure of the *Hs*QPRT-substrate complex, the side chain of R161 in loop L was moved by approximately 9.5 Å outward relative to its position within *Hs*QPRT-open. G165 and G166 of loop L, which is only conserved in mammalian QPRTs (Supplementary Fig. S4), not only participates in the interaction but also provides structural flexibility enabling loop L to move dynamically around the dimer-dimer interface. Loop O of the *Hs*QPRT-open structure eventually opens the loop conformation, showing an approximately 5.3 Å difference compared to that of the substrate complex. Loops L and O were stabilised in an open conformation through hydrophobic loop-loop interactions with those of an adjacent subunit. Structural comparison of the *Hs*QPRT and substrate complex indicates that loop L functions as a lid for substrate binding and that R161 is an allosteric residue that not only functions as a key player in the active site in the presence of a ligand but also as a mediator between the intermolecular interactions to release the product and form a hexamer in the *Hs*QPRT-open structure. This is consistent with the kinetic analysis of the effects of various mutations around the active sites, which showed that the R161A mutation could abolish substrate binding and enzyme activity with a 20% capacity of wild-type^26^. Consequently, substrate binding of one protomer in one dimer caused conformational changes in the adjacent protomer in the other dimer via loops L and O. In addition, changes in the interaction between the two dimers upon substrate binding also supports that *Hs*QPRT exists as a hexamer that induces conformational change in the adjacent two dimers while the substrate binds to one dimer to enable reactant binding. This was further explored and is described in the next section.

The mode of binding of the product NAMN molecule to human QPRT also showed a distinctive structural difference near the dimer-dimer interface compared to that of the reactant QA (Fig. 3c). Two hydroxyl groups of D193 in MolA of *Hs*QPRT-QA showed an ionic interaction with H133′ of MolF. In contrast, D193 in MolA of *Hs*QPRT-NAMN moved by approximately 4.9 Å to form ionic contacts with R189 in MolA, which enabled only one hydroxyl group to interact with H133′ of MolF. These changes in the dimer interactions caused the product complex to have a more relaxed hexamer. The contact areas between the two dimers in *Hs*QPRT-QA and *Hs*QPRT-NAMN were 2127 and 2010 Å^2^, respectively. These results suggest that QA-bound *Hs*QPRT maintains a stable hexamer to mediate the subsequent reaction, such as PRPP binding and phosphoribosyl transfer into QA to generate NAMN. After the reaction, a weaker dimer-dimer interaction between *Hs*QPRT and NAMN compared to QA appeared to be favourable for release of the reaction product. Changes in the interaction between two dimeric subunits were found not only in reactant binding but also in the conversion from the reactant to the product.

### Significance of the N-terminal alpha helix in hexamer formation in human QPRT

**Figure 4:**
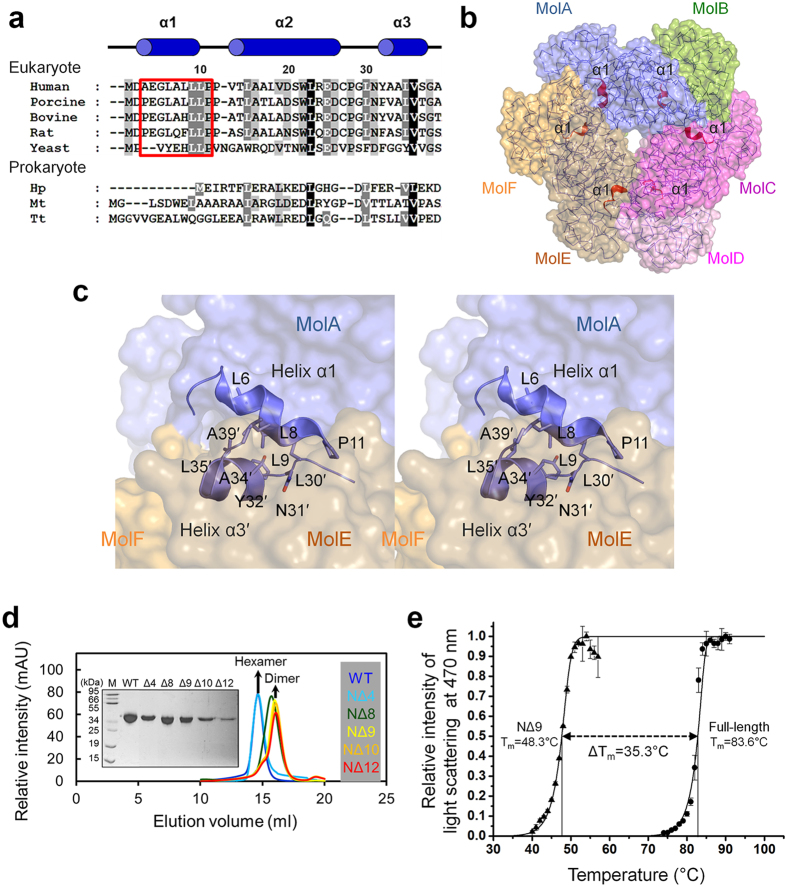
Involvement of helix α1 of *Hs*QPRT in dimer-dimer interaction. (**a**) Multiple sequence alignment of hexameric QPRT homologs. The database and accession code of aligned sequences are indicated in parentheses. *Homo sapiens* QPRT, human (Uniprot code Q15274); *Sus scrofa* QPRT, porcine (PDB code 4I9A); *Bos taurus* QPRT, bovine (NCBI code AAI02551); *Rattus norvegicus* QPRT, rat (NCBI code AAH88177); *Saccharomyces cerevisiae* QPRT, yeast (PDB code 3C2E); *Helicobacter pylori* QPRT, Hp (PDB code 2B7N); *Mycobacterium tuberculosis* QPRT, Mt (PDB code 1QPO); *Thermus thermophilus* QPRT, Tt (PDB code 1 × 1O). Red box indicates N-terminal helix α1. Annotation of helices α1–α3 (blue cylinders) corresponding to *Hs*QPRT. Residue numbers are based on human protein. (**b**) Location of helix α1. Six α1 helices in the hexameric protein are shown as red ribbons. (**c**) Intermolecular interaction between helix α1 and helix α3 of the adjacent dimer (stereographic views). Residues of helix α3 in the adjacent dimer (MolE) are primed. (**d**) Size exclusion chromatographic analysis of *Hs*QPRT in wild-type (blue), NΔ4 (light blue), NΔ8 (green), NΔ9 (yellow), NΔ10 (orange) and NΔ12 (red). The sample state was confirmed using SDS-PAGE. (**e**) Heat aggregation profile of *Hs*QPRT. Time intervals of approximately 2 min (1 °C) are given between data points. Full-length and NΔ9 mutant proteins are labelled as circles and triangles, respectively.

The N-terminal region of eukaryotic QPRTs from yeast to human mainly consists of highly conserved hydrophobic residues such as leucine and proline (Fig. 4a). Although some of the prokaryotic QPRTs such as *Hp*QPRT and *Tt*QPRT appear to exist as hexamers, most exhibit a dimeric configuration. This finding supports the hypothesis that eukaryotic QPRTs maintain the stability of hexamer assembly by generating a hydrophobic interaction with the adjacent dimer within the hexamer structure through their N-terminus.

Interestingly, structures of mammalian QPRTs have unique features compared with that of the yeast enzyme, such as an α-helix of approximately two turns at the N-terminus (helix α1), which is located in the dimer-dimer interface such as loops L and O (Fig. 4b). Consistent with this hypothesis, helix α1 of MolA (A3–P11) in the *Hs*QPRT structure interacts with helix α3′ (Y32′–S37′) in the adjacent dimer (Fig. 4c). The conformation of helix α1 is very similar among apo and complex structures with substrates, the root mean square deviations of which are less than 1.0 Å, as shown in Fig. 3b and Supplementary Figure S5. Irrespective of substrate binding, highly conserved N-terminal hydrophobic residues L6–P11 in MolA form extensive hydrophobic contacts with L30′–A39′ in another subunit of the adjacent dimer, MolE. Because the interface between the dimers is mostly hydrophobic, interactions mediated by hydrophobic residues in helix α1 are highly conserved around mammalian QPRTs and contribute to the formation of the dimer-dimer interface. Hydrophobic interactions in helix α1 in *Hs*QPRT occupy approximately 30% of the total contact area between the two dimers. Thus, two intermolecular interactions, such as MolA-MolE (helix α1-helix α3′) and MolA-MolF (loop L-loop O′), possibly play an essential role in hexamerisation of the *Hs*QPRT structure with sustained dimeric interfaces. To examine the role of hydrophobic residues in the N-terminus of *Hs*QPRT involved in the oligomeric state, we generated serial N-terminal deletion mutants (NΔ4, NΔ8, NΔ9, NΔ10 and NΔ12) (Fig. 4d). NΔ4 eluted as a hexamer that was similar to wild-type, while NΔ8, NΔ9, NΔ10 and NΔ12 were eluted as dimers, indicating that *Hs*QPRT hexamerisation may be dependent on three hydrophobic residues, L8, L9 and P11. These residues are highly conserved throughout mammalian enzymes, presuming that truncated *Hs*QPRT at the N-terminus resembles the dimer found in yeast enzyme. This finding is consistent with the result of the same experiment using full-length *Sc*QPRT and its N-terminal truncated mutant (NΔ10^Sc^) (Supplementary Fig. S6). The full-length enzyme exists as a hexamer, but NΔ10^Sc^ was found to be in the dimeric state, which is consistent with the size distribution from hexameric to dimeric *Hs*QPRT. This indicates that the N-terminal hydrophobic residues of eukaryotic QPRTs are important for hexamer formation.

**Figure 5:**
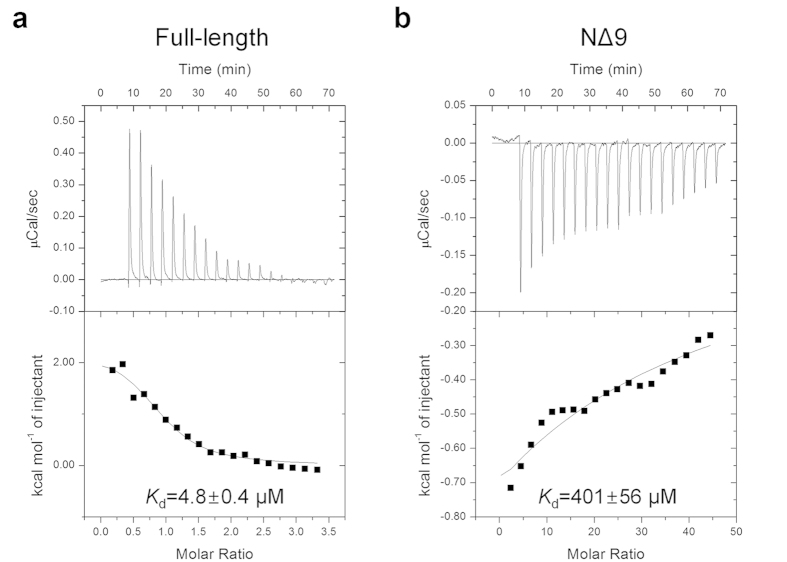
ITC profiles for the binding of QA to *Hs*QPRT. (**a**) ITC data of QA (500 μM) injected into full-length *Hs*QPRT (30 μM) with N (stoichiometry) = 1.0, *K*_d_ = ~4.8 μM, ΔH = ~2.2 kcal/mol and ΔS = ~32 cal/mol. (**b**) ITC data of QA (500 μM) injected into NΔ9 mutant (3 μM) with N = 1.0, *K*_d_ = ~401 μM, ΔH = ~−106 kcal/mol and ΔS = ~−342 cal/mol.

To determine how helix α1 is involved in hexamer stabilisation in *Hs*QPRT, we performed a heat aggregation test using the spectrophotometric method. Interestingly, the half-aggregation temperature of full-length *Hs*QPRT (83.6 °C) was approximately halved for the mutant missing helix α1, which included only nine residues (NΔ9, 48.3 °C) (Fig. 4e). Combined with structural information, these results indicate that elimination of the hydrophobic interaction by helix α1 sufficiently makes *Hs*QPRT sufficiently unstable to maintain the hexameric configuration. However, how conversion of hexameric *Hs*QPRT to a dimeric protein by eliminating helix α1 affects the enzyme function remains unclear, although the NΔ9 mutant has residues forming the intact active site. Additionally, Liu *et al.* reported that the reactant QA binds to *Hs*QPRT prior to PRPP binding^26^. Thus, we evaluated the binding affinity of both the full-length protein (hexamer) and the NΔ9 mutant (dimer) to the first-order reactant QA using isothermal titration calorimetry (ITC) (Fig. 5). The *K*_d_ value between QA and the NΔ9 mutant (401 ± 56 μM) was decreased by approximately 80-fold than that of the full-length protein (4.8 ± 0.4 μM) and was similar to the wild-type *Sc*QPRT (450 ± 30 μM)^27^. The QA binding into full-length *Hs*QPRT is endothermic and entropically driven, suggesting that the dynamicity between the open and closed conformations of the hexameric *Hs*QPRT is important for binding. Such dynamicity is due to the flexible loops formed the dimer-dimer interface conserved only in mammalian QPRT (Supplementary Fig. S4). The dimeric NΔ9 mutant may not have structural flexibility because it lacks the dimer-dimer interaction, which may affect formation of the closed conformation and make the reaction exothermic. Although *Sc*QPRT forms a hexamer, it showed only a closed conformation even though the apo form, which may hinder QA accessibility. This explains why the NΔ9 mutant showed similar binding affinity to *Sc*QPRT. In contrast, full-length *Hs*QPRT stabilised the hexamer, which could form an open conformation (Supplementary Fig. S7). Thus, hexameric *Hs*QPRT enhances QA accessibility, improving binding affinity. The results of the ITC experiment indicated that *Hs*QPRT exists as hexamer to enhance the efficiency of reactant binding by stabilizing the three dynamic dimer-dimer interfaces of hexameric *Hs*QPRT to accommodate the reactant. Moreover, we measured the binding affinity and calculated the Hill coefficient to analyse the cooperativity of binding to the hexameric *Hs*QPRT for QA, phthalic acid, PRPP, phthalic acid with saturated PRPP and PRPP with saturated phthalic acid. We used phthalic acid rather than QA for bi-substrate binding, as the reaction interfered with the affinity measurement in the presence of the two substrates. We calculated the Hill coefficient by analysing the Hill plot. The *K*_d_ value of QA was 4.8 ± 0.4 μM with a Hill coefficient of 1.28 ± 0.09, indicating that QA binds to hexameric apo *Hs*QPRT with positive cooperativity (Fig. 5a and Supplementary Fig. S8). The binding property of phthalic acid to *Hs*QPRT was very similar to that of QA with a *K*_d_ value and Hill coefficient of 5.2 ± 0.2 μM and 1.25 ± 0.02, respectively (Supplementary Fig. S9). Thus, phthalic acid is a good analogue of QA for measuring bi-substrate binding and the cooperativity of substrate binding. We could not measure the binding properties of PRPP likely because of the low binding affinity of PRPP (Supplementary Fig. S10a). The *K*_d_ value of PRPP for the yeast enzyme was reported to be approximately 10 mM, which exceeds the limit of ITC measurement^27^. In order to investigate the binding affinity and cooperativity for the binding of the second substrate, we titrated PRPP under saturated conditions of phthalic acid and *vice versa* (Supplementary Fig. S10,S11). Under phthalic acid saturated conditions, PRPP binding showed positive cooperativity, with a *K*_d_ value and Hill coefficient of 124 ± 5 μM and 1.14 ± 0.01, respectively. These results indicate that PRPP binding affinity increases in the presence of the first-order substrate QA. Cooperativity of the second substrate (PRPP) binding was lower than that of the first substrate (QA). In the reverse experiment, under PRPP saturated conditions, phthalic acid binding affinity dramatically decreased by approximately 6-fold (*K*_d_ = 33.3 ± 3.7 μM) and the positive cooperativity decreased to 1.11 ± 0.01. This result indicates that PRPP inhibits first-order substrate (QA) binding. Based on structural and biochemical evidence, mammalian QPRTs including the human enzyme may exist as a hexamer by the trimerisation of dimers assisted by helix α1, consisting of only nine residues in its N-terminus. Moreover, the hexameric configuration is essential for not only stabilizing the hexameric enzyme through intermolecular hydrophobic interactions between the two dimers independently of substrate binding, but also contributes to cooperative reactant binding.

## Discussion

**Figure 6:**
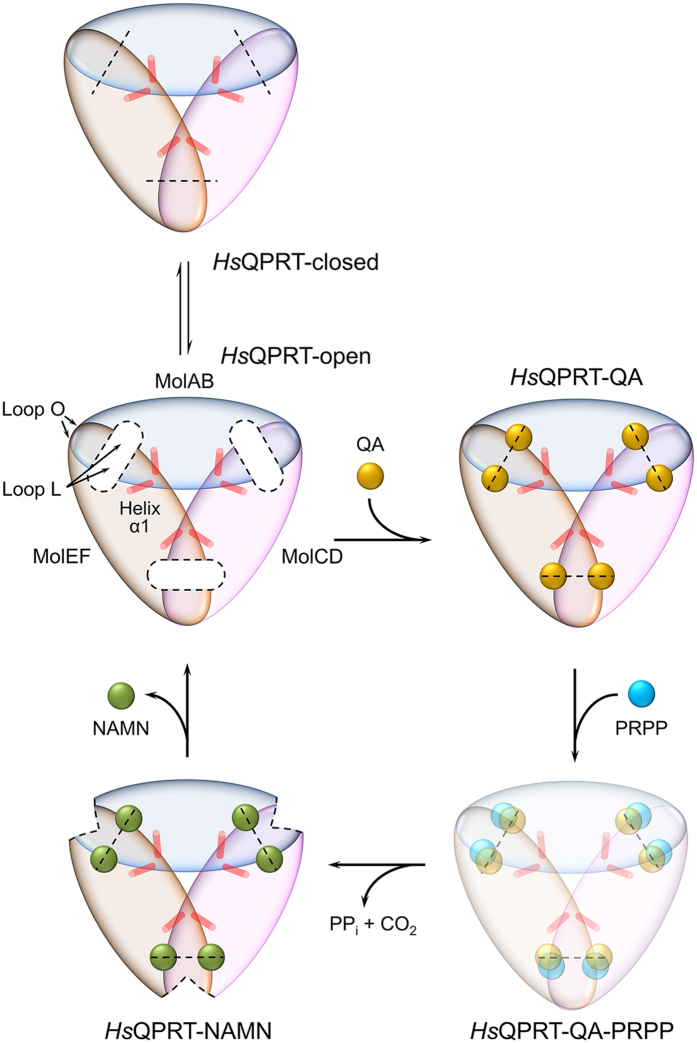
Proposed model of reaction catalysis mediated by *Hs*QPRT. The three dimer-dimer interfaces showing conformational changes are shown as dashed lines. Helices α1 are displayed as red cylinders. QA- and PRPP-bound intermediate state not determined yet is faded (lower right).

In this study, we examined the molecular basis of the unexpected rearrangement of hexamer assembly caused by conformational changes in three dimer-dimer interfaces during the reaction process, as well as hexamer stabilisation of *Hs*QPRT by the N-terminal short helix α1. Thus, we propose a reaction model for *Hs*QPRT (Fig. 6). Although the apo structure of *Hs*QPRT showed dynamic movement between the open and closed conformations in its active site and its dimer-dimer interface, it can exist as a hexamer through extensive hydrophobic contacts with helix α1. Some pentosyltransferases (EC 2.4.2) adopt a hexameric configuration through the trimerisation of dimers, such as nucleoside phosphorylases (PDB codes 4E1V, 4D8Y and 4R2X)^35^,^36^,^37^; however, it is unusual for the short α helix of *Hs*QPRT to contribute to hexamer stability while its flexible loops show dynamic conformational changes. First, loops L and O of *Hs*QPRT were closed upon QA binding. Next, PRPP bound to the carboxylate moiety of QA to produce NAMN. Because PRPP is less stable than QA because of its highly reactive pyrophosphate group and therefore cannot remain in the active site for a long period of time, it is rational that QA can occupy the active site before PRPP enters. This is also consistent with the results of a previously reported kinetic analysis performed by Liu *et al.*^26^. As the reaction progresses, *Hs*QPRT changes the conformation of loop O in the dimer-dimer interface, making the hexamer slightly less stable. Once an NAMN molecule is generated after the phosphoribosyl transfer reaction, *Hs*QPRT fully unfastens its active site and regains the apo form, which can adopt open and closed states. The dynamicity of these dual conformations may provide the driving force to release NAMN.

NAD^+^ plays a key role in cell survival and growth, particularly by maintaining redox homeostasis and cellular energy generation. Given the necessity of excessive NAD^+^ levels in tumour cells to maintain their rapid growth, inhibiting NAD^+^ biosynthesis is an attractive target for the development of anticancer agents. In this context, extensive efforts have been exerted to design compounds that induce apoptosis of tumour cells with NAD^+^ depletion by inhibiting enzymes associated with NAD^+^ synthesis. However, the design of anticancer agents that inhibit NAD^+^ biosynthesis has only focused on the salvage pathway, in which nicotinamide is a precursor. For instance, FK866 (APO866), a low-molecular-weight compound, has been reported to exhibit potent efficacy in a series of cancers such as breast, gastric and liver cancer, inhibiting nicotinamide phosphoribosyltransferase, a rate-limiting enzyme in the salvage pathway of NAD^+^ synthesis, and is currently being examined phase II clinical trials. Importantly, a recent study suggested that QPRT is highly involved in *de novo* NAD^+^ synthesis in glioma in which approximately 30% of tumours are derived from the brain and central nervous system and 80% from all malignant brain tumours^20^. Interestingly, QPRT is exclusively expressed in the World Health Organization high-grade (WHO grade III–IV) glioma and produces NAD^+^ using QA taken from normal microglial cells. This enables glioma to survive under conditions of oxidative stress and NAD^+^ depletion caused by therapeutic approaches, including irradiation and alkylating agent treatment^21^. In this study, we determined that *Hs*QPRT structures are present in apo and substrate-bound forms. In particular, the novel open conformation first observed in our apo structure provides information that can be used in the development of anticancer agents for treating high-grade glioma. Thus, agents targeting QPRT in glioma should be developed using information from the structures. Despite the substitution of QA with phthalate, which has an inhibitory effect in response to *Hs*QPRT, the low selectivity related to its simple structure makes NAMN more useful as a starting point for developing a novel anti-glioma agent.

**Figure 7:**
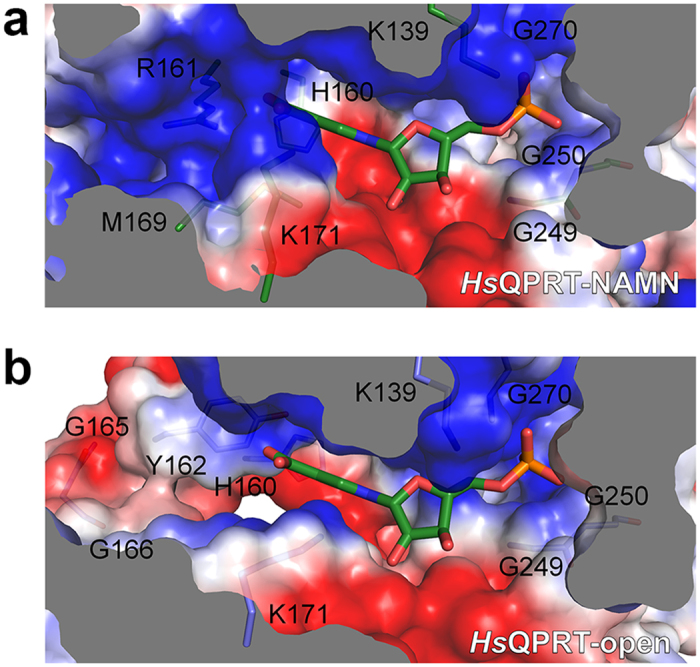
Structural comparison of substrate binding sites. Surface representations of substrate binding pockets between *Hs*QPRT-NAMN ((**a**), green) and *Hs*QPRT-open ((**b**), blue). Positive and negative surface charges of the protein are coloured in blue and red, respectively. The NAMN molecule is shown as sticks.

To design an NAMN-based QPRT inhibitor with higher specificity compared to substrates with extra moieties, NAMN was placed in the *Hs*QPRT-open structure, demonstrating novel conformational changes in the NAMN binding site compared with the *Hs*QPRT-NAMN structure (Fig. 7). No hindrance was observed when NAMN was placed in the *Hs*QPRT-open structure. The orientations of the residues interacting with the ribose and phosphate groups were nearly the same in both *Hs*QPRT-open and *Hs*QPRT-NAMN structures such that the structures did not have to be further optimised. However, there was a difference in the interaction with the nicotinate moiety between the two QPRT structures of both structures. In *Hs*QPRT-NAMN, nicotinate interacted with H160, R161, M169 and K171 to form a cavity that blocked another interaction with nicotinate, specifically through the close ionic contact of the R161 in loop L and K171 with 3-caboxylate in nicotinate. In contrast, the ligand binding cavity remained open in the *Hs*QPRT-open structure because of the conformational changes in both R161 and K171. This cavity consisted of two positively charged patches wrapped around 3-carboxylate and one negatively charged patch. Modification of the carboxylate group in the NAMN molecule with polar moieties connected in the aliphatic chain generated an additional hydrogen bond with the cavities in the *Hs*QPRT-open structure. These findings suggest that the scaffolds are promising for developing a novel anti-glioma agent that selectively targets the first structure of *Hs*QPRT-open with unique conformational properties of loops in the substrate binding site around the dimer-dimer interface.

In summary, we reported the structures of *Hs*QPRT associated with *de novo* NAD biosynthesis in the apo form with a novel open conformation and in complex with QA or NAMN as a reactant or product. We determined the physiological relevance of *Hs*QPRT-QA compared with tartrate as a QA mimic. Apo and complex structures bound to substrate revealed that loops L and O, located in the dimer-dimer interface of hexameric *Hs*QPRT, altered their conformations upon substrate binding and played a key role in reactant binding by changing the interaction between the two dimers. Moreover, to investigate the function of N-terminal hydrophobic residues in eukaryotic QPRTs, we performed structural comparisons and several biochemical studies, including size exclusion chromatography, a heat aggregation test and ITC measurements. We propose that conserved hydrophobic residues in helix α1 stabilise the hexameric configuration of eukaryotic QPRTs by reinforcing the dimer-dimer interaction. Based on structural and biochemical studies, we determined that *Hs*QPRT exists and functions as a hexamer to bind the reactant cooperatively and to maintain its stability. Based on the extra cavity of *Hs*QPRT-open compared with *Hs*QPRT-NAMN, we also suggest a strategy for drug development targeting *Hs*QPRT to treat malignant glioma with high specificity. The results of this study provide biological information related to the functional insights of *Hs*QPRT and anticancer agent design.

## Methods

### Protein expression and purification

DNA encoding full-length *Hs*QPRT (residues 1–297) was amplified from human cDNA (GenBank code 30583300) by polymerase chain reaction using a PCR system 2720 thermocycler (Applied Biosystems, Foster City, CA, USA) and subcloned into the *Nde*I and *Xho*I sites of the pET21a (Novagen, Madison, WI, USA) expression vector. To scale up the over-expressed cells, *Hs*QPRT protein was expressed in *Escherichia coli* BL21 (CodonPlus) strain at 37 °C with 1 mM isopropyl-D-thiogalactoside (Pharmacia, Uppsala, Sweden) for 6 h after induction. After disrupting the cells by sonication, the cell lysate was harvested by low-speed centrifugation at 4 °C and loaded onto a gravity-flow column (Bio-Rad, Hercules, CA, USA), which was packed with nickel-nitriloacetic acid agarose resin (Peptron, Daejeon, Korea) and pre-equilibrated with lysis buffer (50 mM NaH_2_PO_4_, pH 7.5 and 300 mM NaCl). After washing with lysis buffer, the *Hs*QPRT protein was eluted using lysis buffer additionally containing 250 mM imidazole. The protein was concentrated using a Centriprep YM-3 (Millipore, Billerica, MA, USA) and then subjected to Hiload 16/60 Superdex 200 prep grade (GE Healthcare, Little Chalfont, UK) size exclusion chromatography equilibrated with 20 mM HEPES-NaOH, pH 7.5 and 100 mM KCl. The fractions containing the recombinant protein were confirmed using SDS-PAGE and subsequently pooled and concentrated to 15 mg/mL for initial crystallisation. The N-terminal truncated *Hs*QPRTs (NΔ4, NΔ8, NΔ9, NΔ10 and NΔ12) were prepared using the same procedures as used for the full-length protein. All proteins were obtained in > 98% purity as confirmed by SDS-PAGE. Concentrations for *Hs*QPRT were determined using the following theoretical extinction coefficients (26470 M^−1^ cm^−1^) with PROTPARAM^38^ and were calculated with an additional leucine, glutamate and hexahistidine tag in the C-terminus.

### Crystallisation and data collection

The crystallisation of apo *Hs*QPRT has been previously described^39^. Briefly, plate-like crystals of apo *Hs*QPRT were grown at 21 °C in 2 μL hanging drops consisting of equal volumes of protein solution (15 mg/mL) and mother liquor consisting of 100 mM MES-NaOH, pH 5.0, 7–15% (w/v) PEG MME 2K and 10 mM potassium thiocyanate. The crystals of *Hs*QPRT-QA were grown under mother liquor consisting of 100 mM TRIS-HCl, pH 8.0, 6–10% (w/v) PEG 8K, 100 mM magnesium acetate and 6 mM PRPP (1:10 molar ratio). Next, the crystal was soaked with 6 mM QA for 15 h. Although we performed co-crystallisation of QA only, this crystal diffracted poorly. In addition, we also collected datasets of the co-crystal of *Hs*QPRT-PRPP and obtained the electron density, but we could not find electron density corresponding to PRPP. PRPP may contribute in maintaining the crystal packing, although PRPP was not detected in the electron density map. During the process of soaking in QA, it is likely that PRPP was exhausted by hydrolysis and the reaction observed was QA binding at the active site. *Hs*QPRT-NAMN was co-crystallised under a reservoir solution under the same conditions for crystallisation of the apo enzyme except 100 mM sodium acetate, pH 5.0, rather than MES-NaOH was used with protein-NAMN (1:10 molar ratio) pre-incubated at 4 °C for 15 h. The crystals grew to a maximum size of 0.3 × 0.3 × 0.1 mm over one week. The crystals were cryoprotected in the reservoir solution supplemented with 25% (v/v) glycerol and flash-frozen under N_2_ gas at 95 K. Native datasets of apo crystals were collected using an ADSC Quantum 210 CCD detector at beamline NW12A in PF-AR of the Photon Factory, Japan at a wavelength of 1.0000 Å. Datasets of *Hs*QPRT-QA and *Hs*QPRT-NAMN were collected using an ADSC Quantum 315 CCD detector at beamline 5C in the Pohang Accelerator Laboratory, Korea at a wavelength of 0.9795 Å.

### Structure determination

The apo crystal belonged to the space group *P*2_1_ with unit cell dimensions of *a* = 76.2, *b* = 137.1, *c* = 92.7 Å and *β* = 103.8°. All datasets were processed and scaled using HKL2000^40^. The asymmetric unit contained one hexamer (MolA–MolF) with a Matthews coefficient of 2.46 Å Da^-1^ and solvent content of 49.8%, as calculated using the Matthews coefficient^41^ in the CCP4i 6.5.0. suite^42^. The apo *Hs*QPRT structure was determined at 2.8 Å resolution using the molecular replacement method with the Phaser^43^ program using the structure of the *Hs*QPRT-tartrate complex (PDB code 2JBM) as a search model. Next, refinement cycles were performed using the REFMAC5^44^ program in the CCP4i 6.5.0. suite^42^. No electron density was observed in some regions of each molecule (residues 159–165 and 192–197 in MolD and 159–166 in MolE). Phases of *Hs*QPRT-QA or *Hs*QPRT-NAMN were obtained by molecular replacement with Phaser^43^ using the *Hs*QPRT-tartrate complex as a template. Coordinates of nine QA and twelve NAMN molecules were obtained from RCSB Ligand Expo (http://ligand-expo.rcsb.org) and placed into the density corresponds to them. Multiple rounds of refinement were performed using REFMAC5^44^ to final 2.8 (apo), 3.1 (*Hs*QPRT-QA) and 2.6 (*Hs*QPRT-NAMN) Å resolutions, respectively. The models were rebuilt manually using COOT^45^. Final crystallographic *R*_work_ and *R*_free_ values were 20.1 and 25.9% (*Hs*QPRT-open), 18.5 and 23.9% (*Hs*QPRT-QA) and 21.2 and 21.3% (*Hs*QPRT-NAMN). The electron density map of *Hs*QPRT-open and the simulated annealing omit map of *Hs*QPRT-QA and *Hs*QPRT-NAMN showed clear density for two loops (loops L and O) and the substrate molecules (QA and NAMN) bound to the protein (Supplementary Fig. S12,S13). The Ramachandran plots, which were calculated using the program PROCHECK^46^, showed nearly no residues with torsion angles in forbidden areas. Detailed refinement statistics are provided in Table 1. All molecular graphics were generated using PyMOL (The PyMOL Molecular Graphics System, version 1.3, Schrödinger, LLC, New York, NY, USA).

### Size distribution analysis

To determine the sizes of the various constructs, we measured the oligomeric state of *Hs*QPRT and its N-terminal truncated forms (NΔ4, NΔ8, NΔ9, NΔ10 and NΔ12) using Superdex 200 10/300 GL (GE Healthcare) size exclusion chromatography, which was calibrated using standard proteins, including ferritin (440 kDa), catalase (232 kDa) and bovine serum albumin (66 kDa) equilibrated with 20 mM HEPES-NaOH, pH 7.5 and 100 m*M* KCl. To compare the size distribution between *Hs*QPRT and its mutants to other eukaryotic QPRT proteins, we prepared *Sc*QPRT and its N-terminal truncated form (NΔ10) using the same procedures as *Hs*QPRT and then analysed the purification patterns of *Sc*QPRT and NΔ10 using Superdex 200 10/300 GL (GE Healthcare) size exclusion chromatography.

### Heat aggregation test

To evaluate the stability of *Hs*QPRT, the half aggregation temperature was determined spectrophotometrically using a previously described method^33^. The reaction mixture contained 25 mM HEPES-NaOH, pH 7.5, 100 mM KCl and 0.2 mg/mL of *Hs*QPRT (full-length and NΔ9). To measure the aggregation profile, the A_470_ of the protein solution was monitored while increasing the temperature at a rate of approximately 1 °C per 2 min. For each sample, experiments were performed in triplicate.

### Isothermal titration calorimetry

ITC measurements were carried out on a VP-ITC Microcalorimeter (Microcal, Northampton, MA, USA). Full-length and NΔ9 *Hs*QPRT and QA were dissolved in 25 mM HEPES-NaOH, pH 7.5, 100 mM KCl and were degassed for 10 min using a ThermoVac (Microcal). To investigate the QA binding properties of the hexamer and dimer, *Hs*QPRT was used as a titrant in the sample cell at a concentration of 30 μM (full-length) or 3 μM (NΔ9), while QA of 500 μM was loaded into the syringe. To investigate cooperativity, full-length *Hs*QPRT at a concentration of 20–30 μM was used as a titrant in the sample cell, while 800 μM phthalic acid or 2 mM PRPP was loaded into the syringe. To generate the saturated condition, additional phthalic acid or 200–300 μM of PRPP (1:10 molar ratio) was added to the sample cell. Experiments were performed at least in duplicate using the following parameters: temperature, 25 °C; reference power, 5 μcal/s; injection volume, 2 μL first injection followed by 15 μL for the remaining 19 injections; spacing between injections, 200 s. Data were analysed by Origin 7 software provided by the manufacturer with curves fitted with a one set of site models. The Hill Plot was constructed using previously described method by Dam *et al.*^47^.

## Additional Information

**Accession codes**: The coordinates and structure factors for HsQPRT-open, HsQPRT-QA and HsQPRT-NAMN have been deposited in the RCSB Protein Data Bank with the accession codes 5AYX, 5AYY and 5AYZ.

**How to cite this article**: Youn, H.-S. *et al.* Structural Insights into the Quaternary Catalytic Mechanism of Hexameric Human Quinolinate Phosphoribosyltransferase, a Key Enzyme in *de novo* NAD Biosynthesis. *Sci. Rep.* **6**, 19681; doi: 10.1038/srep19681 (2016).

## Supplementary Material

Supplementary Information

## Figures and Tables

**Table 1:** Data collection and refinement statistics.

Dataset	*Hs*QPRT-open	*Hs*QPRT-QA	*Hs*QPRT-NAMN
**Data Collection**			
X-ray source	PF-NW12A	PAL-5C	PAL-5C
Wavelength (Å)	1.0000	0.9795	0.9795
Space group	*P*2_1_	*P*3_2_21	*P*2_1_
Unit cell dimensions (Å, °)	a = 76.2	a = 174.2	a = 109.2
	b = 137.1	b = 174.2	b = 101.2
	c = 92.7	c = 211.7	c = 151.6
	β = 103.8	γ = 120	β = 92.5
Resolution (Å)	48–2.80 (2.87–2.80)	45–3.09 (3.15–3.09)	44–2.60 (2.64–2.60)
Total reflections	196103	450339	669832
Unique reflections	42775	64323	101429
Completeness (%)	97.1 (94.2)	98.9 (96.3)	99.2 (99.9)
*R*_merge_ a (%)	6.4 (31.6)	13.7 (62.6)	7.8 (41.5)
Multiplicity	4.6	6.9	6.6
Wilson B-factor	61.2	58.3	29.9
I/σ (I)	18.3 (4.8)	10.9 (4.7)	8.4 (5.1)
**Refinement**			
Resolution (Å)	48–2.80 (2.90–2.80)	45–3.09 (3.20–3.09)	44–2.60 (2.69–2.60)
*R*_work_ total (%)	20.1 (25.9)	18.5 (26.0)	21.2 (30.0)
*R*_free_^b^ total (%)	25.9 (35.6)	23.9 (33.6)	21.3 (33.8)
R.m.s. bond lengths (Å)	0.010	0.009	0.009
R.m.s. bond angles (°)	1.7	1.4	1.1
No. of protein atoms	12480	18972	25296
No. of ligand atoms		108	264
No. of water atoms	19	8	254
Mean B-factor (Å^2^)	64.0	48.0	32.0
Protein (Å^2^)	64.7	48.9	32.2
Ligand/ion (Å^2^)		52.0	29.0
Water (Å^2^)	46.5	34.5	28.7
Ramachandran plot			
Most favored (%)	90.0	91.9	94.6
Additional allowed (%)	9.0	7.6	4.9
Generously allowed (%)	0.3	0.5	0.4
Disallowed (%)	0.1	0.0	0.0

Values in parentheses are for the highest resolution shell.

^a^*R*_merge_ = ∑_*hkl*_∑_*i*_|*I*_*i*_(*hkl*) −<*I*(*hkl*)>|/∑_*hkl*_ ∑_*i*_ *I*_*i*_(*hkl*), where *I*_*i*_(*hkl*) is the intensity of the *i*th observation of reflection *hkl* and <*I*(*hkl*)> is the average intensity of reflection *hkl*.

^b^*R*_free_ calculated with 5% of all reflections excluded from refinement stages using high resolution data.
