# Supplementary material for: Structural Insights into the Quaternary Catalytic Mechanism of Hexameric Human Quinolinate Phosphoribosyltransferase, a Key Enzyme in de novo NAD Biosynthesis
Source: Sci Rep. 2016 Jan 25;6:19681. doi: 10.1038/srep19681 (PMC4726147; doi:10.1038/srep19681)
Supplement: Supplementary Information [file srep19681-s1.pdf]

Supplementary Information

Structural Insights into the Quaternary Catalytic Mechanism of Hexameric Human Quinolinate Phosphoribosyltransferase, a Key Enzyme in *de novo* NAD Biosynthesis

Hyung-Seop Youn[1,2], Tae Gyun Kim[1,2], Mun-Kyoung Kim[1], Gil Bu Kang[1], Jung Youn Kang[1,2], Jung-Gyu Lee[1,2], Jun Yop An[1,2], Kyoung Ryoung Park[1,2], Youngjin Lee[1,2], Young Jun Im[3], Jun Hyuck Lee[4,5] & Soo Hyun Eom[1,2,6,*]

[1]School of Life Sciences, Gwangju Institute of Science and Technology, Gwangju 500-712, South Korea. [2]Steitz Center for Structural Biology, Gwangju Institute of Science and Technology, Gwangju 500-712, South Korea. [3]College of Pharmacy, Chonnam National University, Gwangju 500-757, South Korea. [4]Division of Polar Life Sciences, Korea Polar Research Institute, Incheon 406-840, South Korea. [5]Department of Polar Sciences, Korea University of Science and Technology, Incheon 406-840, South Korea. [6]Department of Chemistry, Gwangju Institute of Science and Technology, Gwangju 500-712, South Korea.

[*]Corresponding author. E-mail address: eom@gist.ac.kr

Supplementary Results

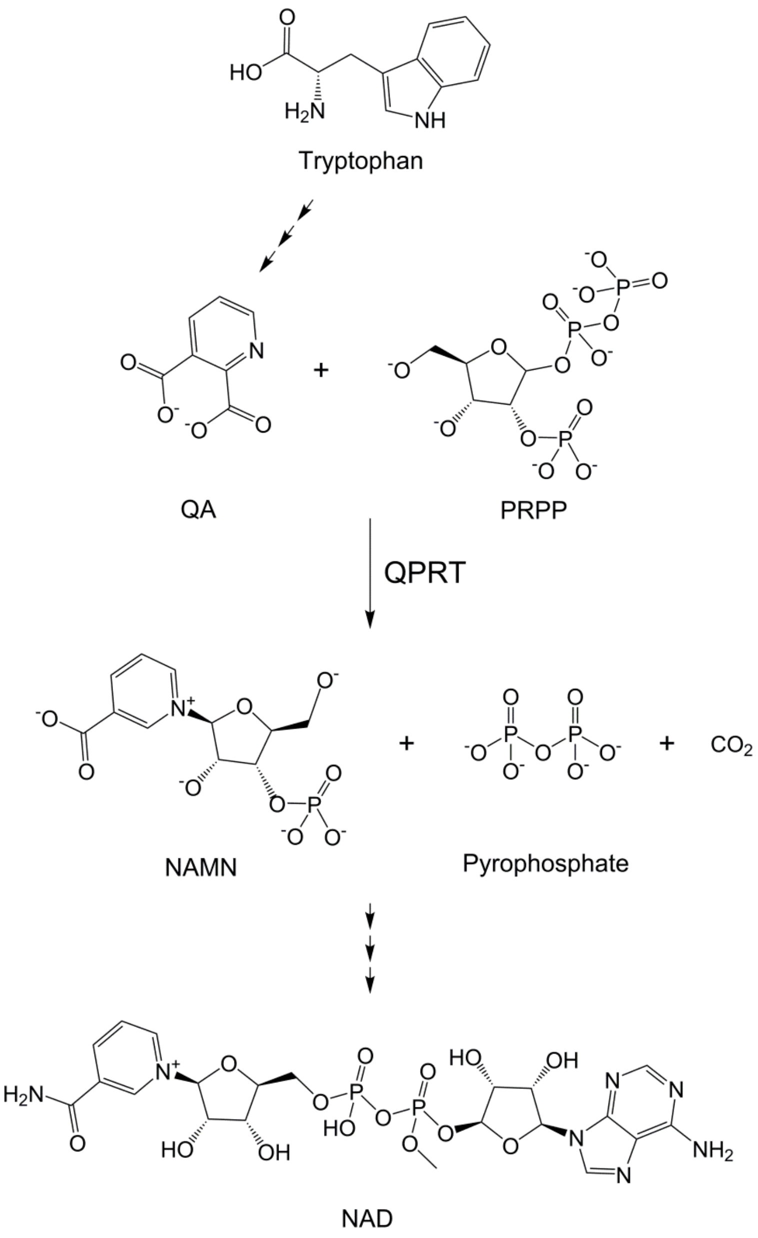

Supplementary Figure S1. Reaction catalyzed by QPRT.

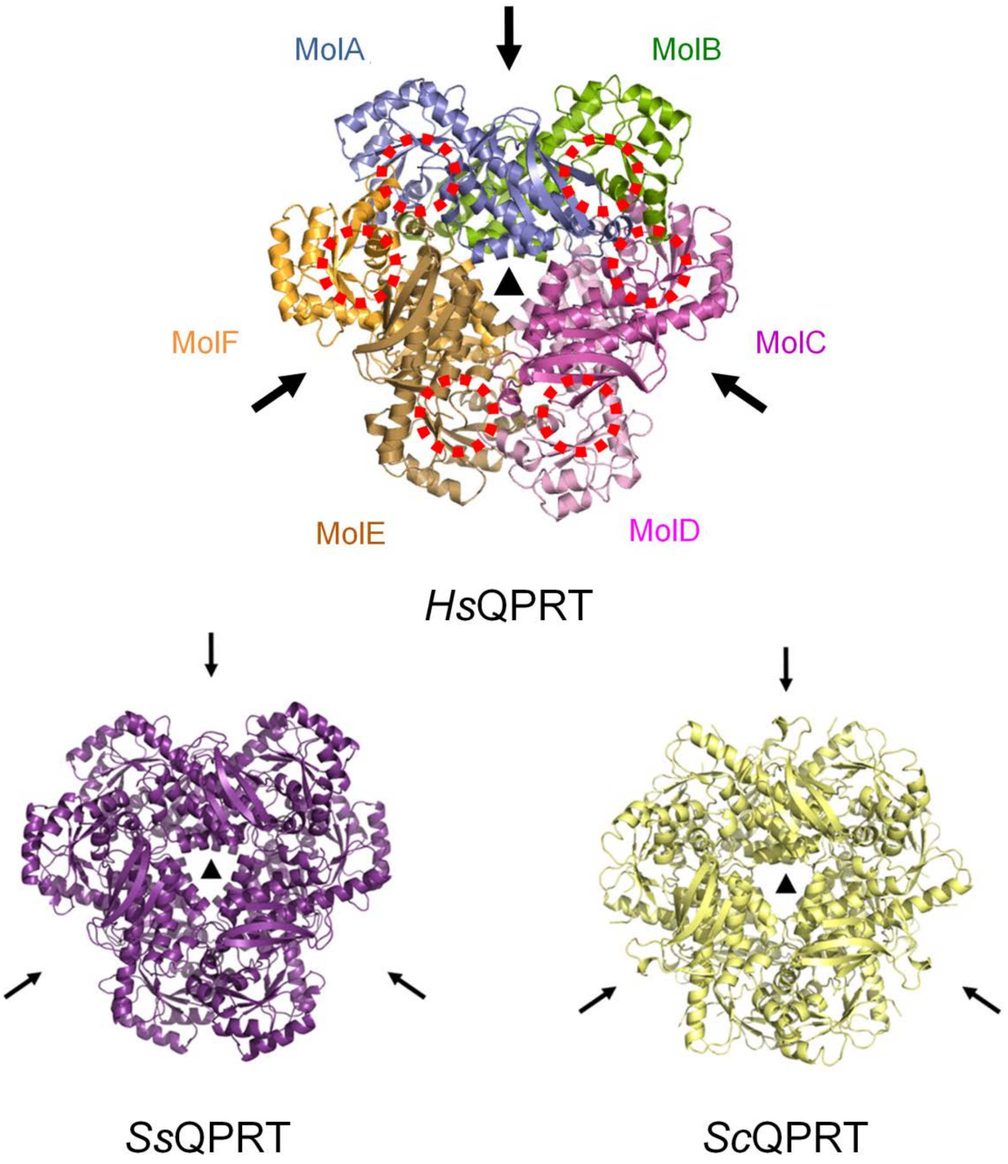

Supplementary Figure S2. Hexameric or dimeric biological units of QPRTs from human and other eukaryotes (*Sus scrofa*, *Ss*; *Saccharomyces cerevisiae*, *Sc*). The two- and three-fold axes of the hexamer are indicated as arrows and triangle, respectively. Active sites in *Hs*QPRT are indicated as dashed circles.

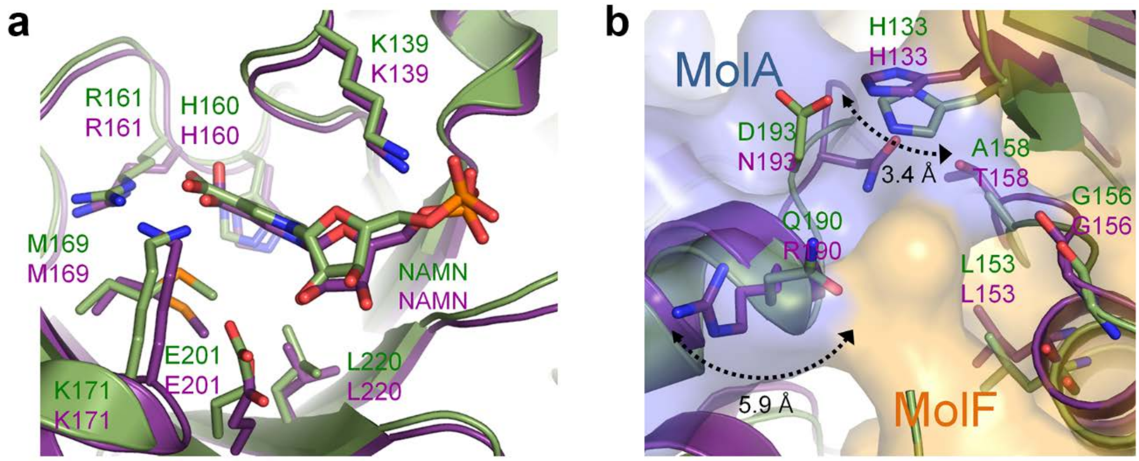

Supplementary Figure S3. Structural comparison of NAMN complexes of human (green) and porcine (purple) QPRTs in the NAMN binding site (a) and dimer-dimer interface (b). The movements are shown as two-sided arrows.

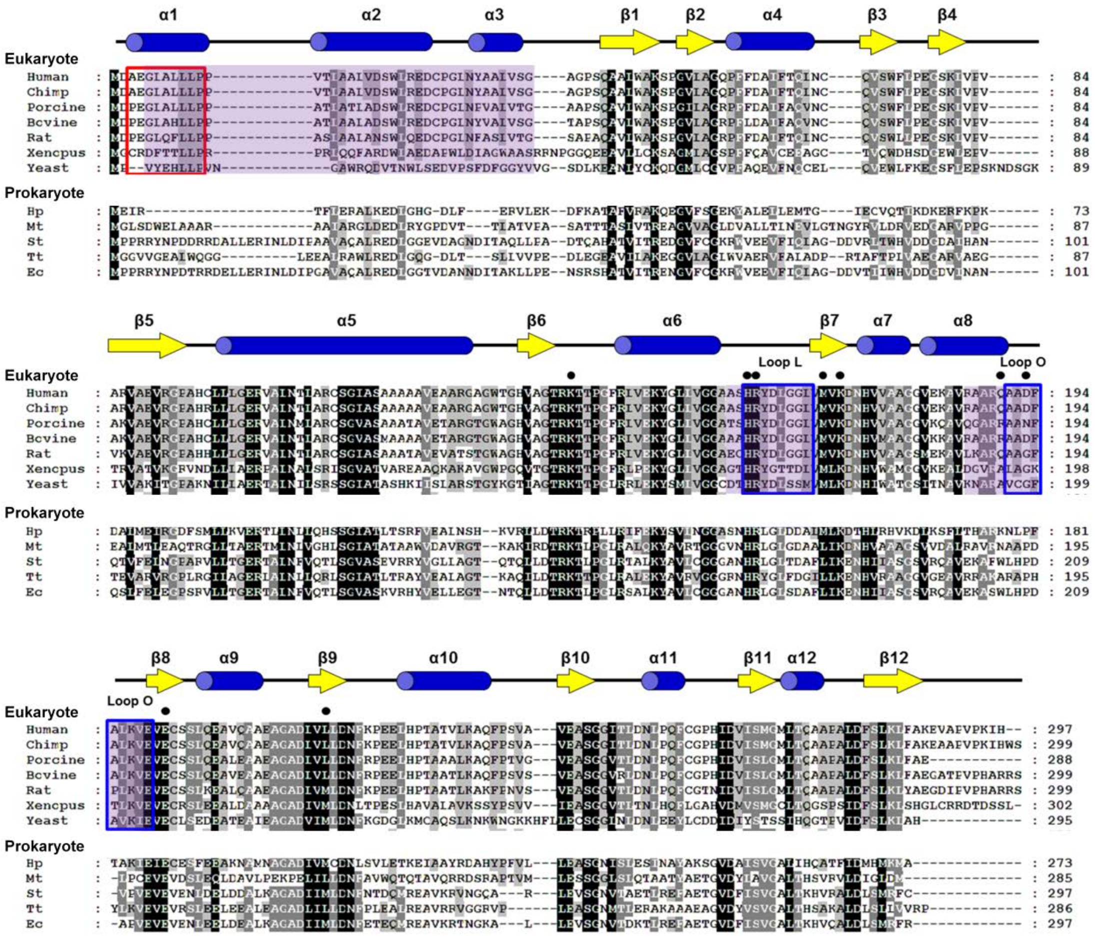

Supplementary Figure S4. Multiple sequence alignment of *Hs*QPRT homologs. Conserved residues are indicated as white letters in the black and gray background. The blue cylinders and yellow arrows represent α-helices and strands of human enzyme, respectively. The black lines indicate loops. The accession number and database of aligned sequences are indicated in parentheses: *Homo sapiens* QPRT, Human (Uniprot code Q15274); *Pan troglodytes* QPRT, Chimp (Uniprot code K7ACA5); *Sus scrofa* QPRT, porcine (PDB code 4I9A); *Bos taurus* QPRT, Bovine (NCBI code AAI02551); *Rattus norvegicus* QPRT, Rat (NCBI code AAH88177); *Xenopus laevis* QPRT, Xenopus (Uniprot code Q6AZR1); *Saccharomyces cerevisiae* QPRT, Yeast (PDB code 3C2E); *Helicobacter pylori* QPRT, Hp (PDB code 2B7N); *Mycobacterium tuberculosis* QPRT, Mt (PDB code 1QPO); *Salmonella typhimurium* QPRT, St (Uniprot code P30012); *Thermus thermophilus* QPRT, Tt (PDB code 1X1O); *Escherichia coli* QPRT, Ec (Uniprot code P30011). Blue boxes indicate residues of loop L and loop O involved in intermolecular interactions among MolA-MolF. The red box indicates the N-terminal helix α1. Residues involved in the active site are indicated as dots in the upper side. Regions involved in the dimer-dimer interface of *Hs*QPRT are shaded in purple.

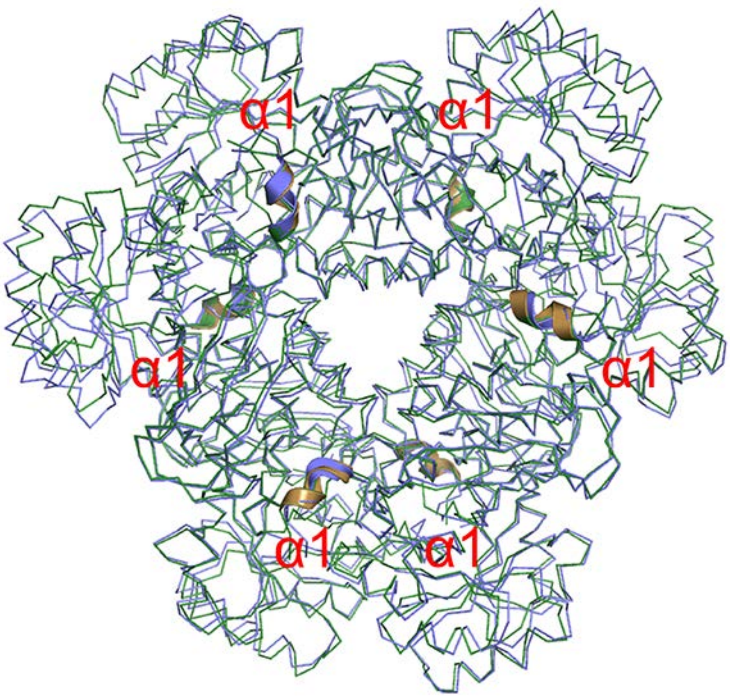

Supplementary Figure S5. Structural comparison of helix α1 of *Hs*QPRT-open (blue), *Hs*QPRT-QA (brown) and *Hs*QPRT-NAMN (green). Helix α1 are shown as cartoon diagrams.

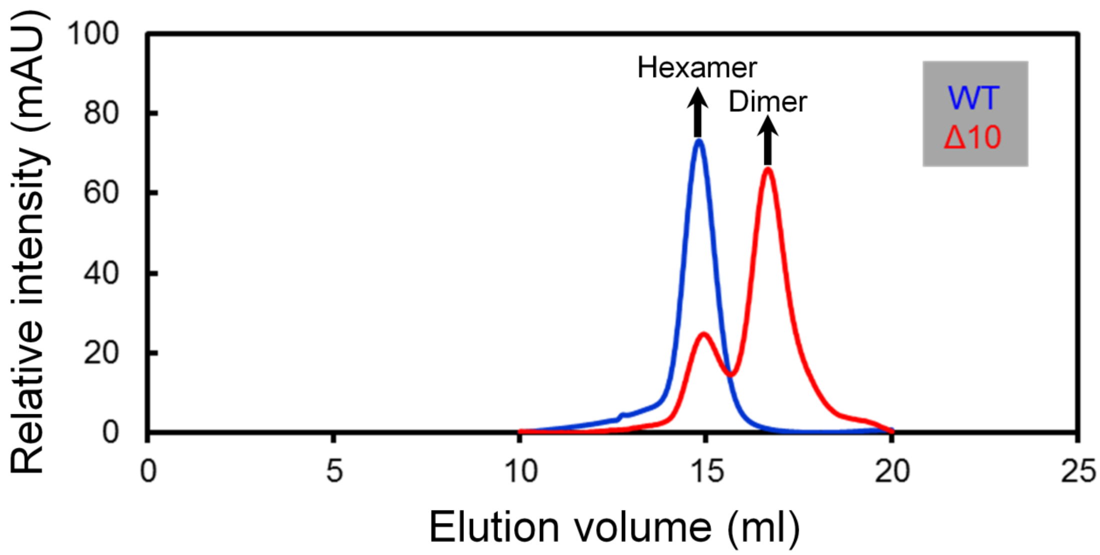

Supplementary Figure S6. Relatively compared size exclusion chromatographic profiles of wild-type of *Sc*QPRT (blue) and its mutant (NΔ10^{Sc}, red). Blue and red peaks approximately correspond to a hexamer and dimer, similar to wild-type *Hs*QPRT and its mutant NΔ12, respectively.

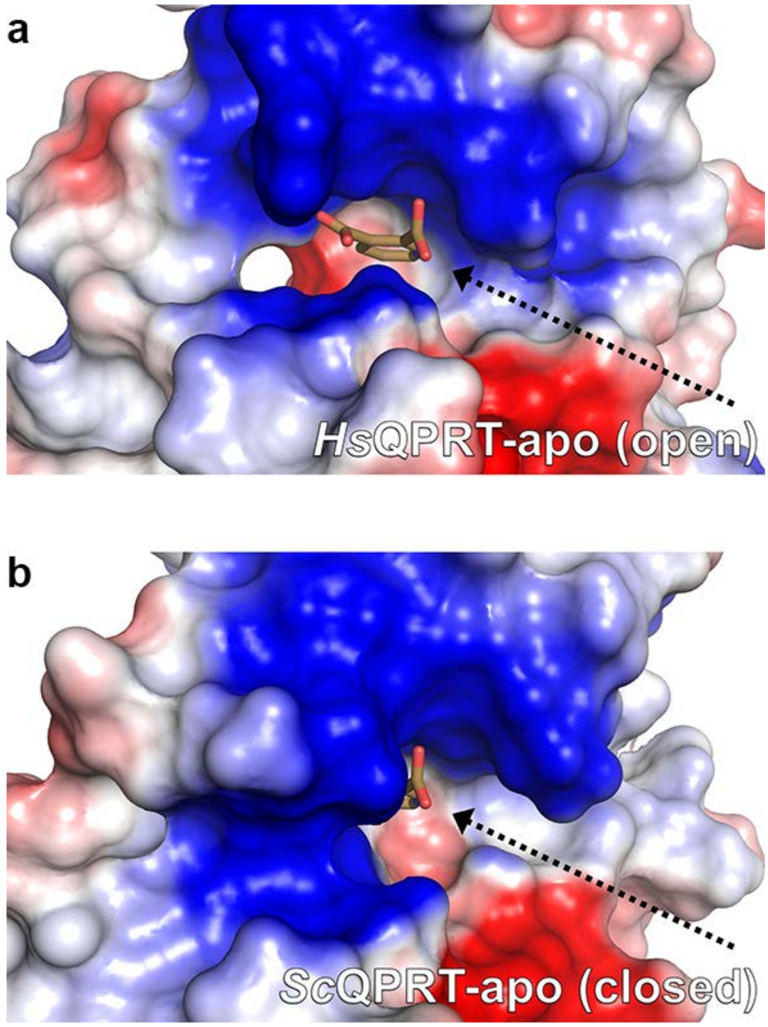

Supplementary Figure S7. Surface representations of QA binding pockets between *Hs*QPRT-open (a) and *Sc*QPRT-apo (b). Positive and negative surface charges of the protein are coloured in blue and red, respectively. The QA molecule (brown stick) is extracted from the *Hs*QPRT-QA structure. Expected directions of QA access are shown as dashed arrows.

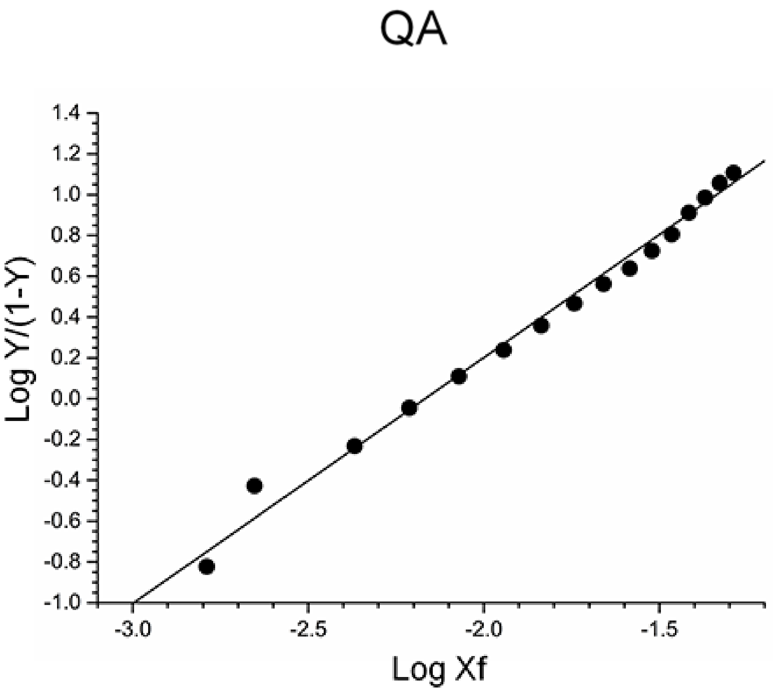

Supplementary Figure S8. Hill plot of QA binding to *Hs*QPRT derived from the ITC data.

Phthalic acid

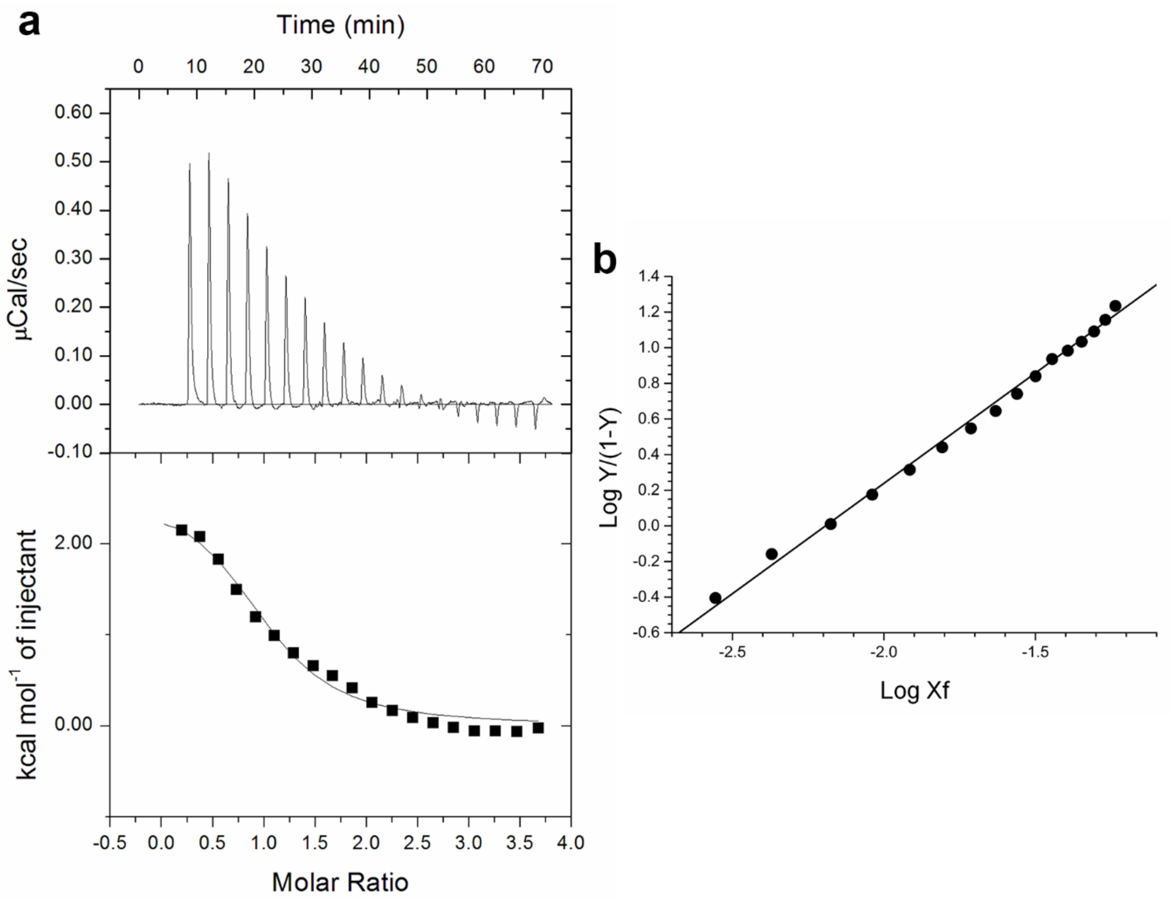

Supplementary Figure S9. (a) ITC profile for the binding of phthalic acid (500 μM) to *Hs*QPRT (30 μM) with N (stoichiometry) = 1.0, K_d = ~5.2 μM, ΔH = ~2.4 kcal/mol and ΔS = ~33 cal/mol. (b) Hill plot derived from the ITC data.

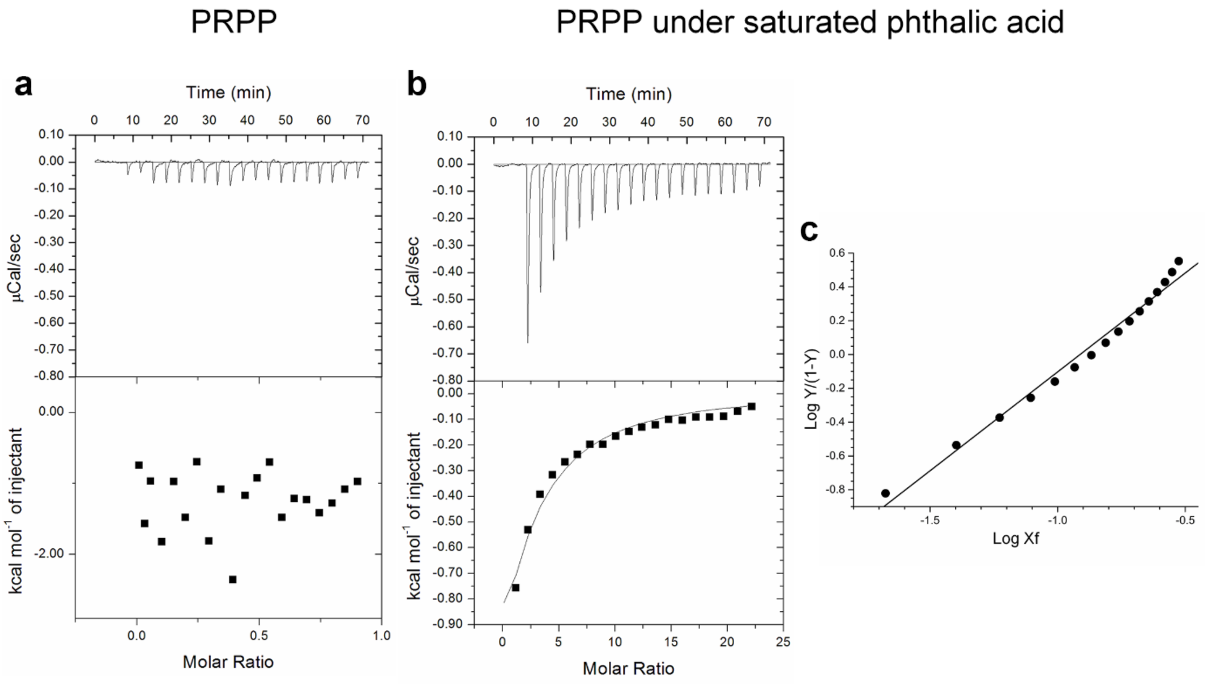

Supplementary Figure S10. (a) ITC profile for the binding of PRPP (2 mM) to *Hs*QPRT (20 μM). (b) ITC profile for the binding of PRPP (2 mM) to *Hs*QPRT (20 μM) under saturated phthalic acid (200 μM) with N (stoichiometry) = 1.0, K_d = ~124 μM, ΔH = ~-6.7 kcal/mol and ΔS = ~-4.8 cal/mol. (c) Hill plot derived from the ITC data.

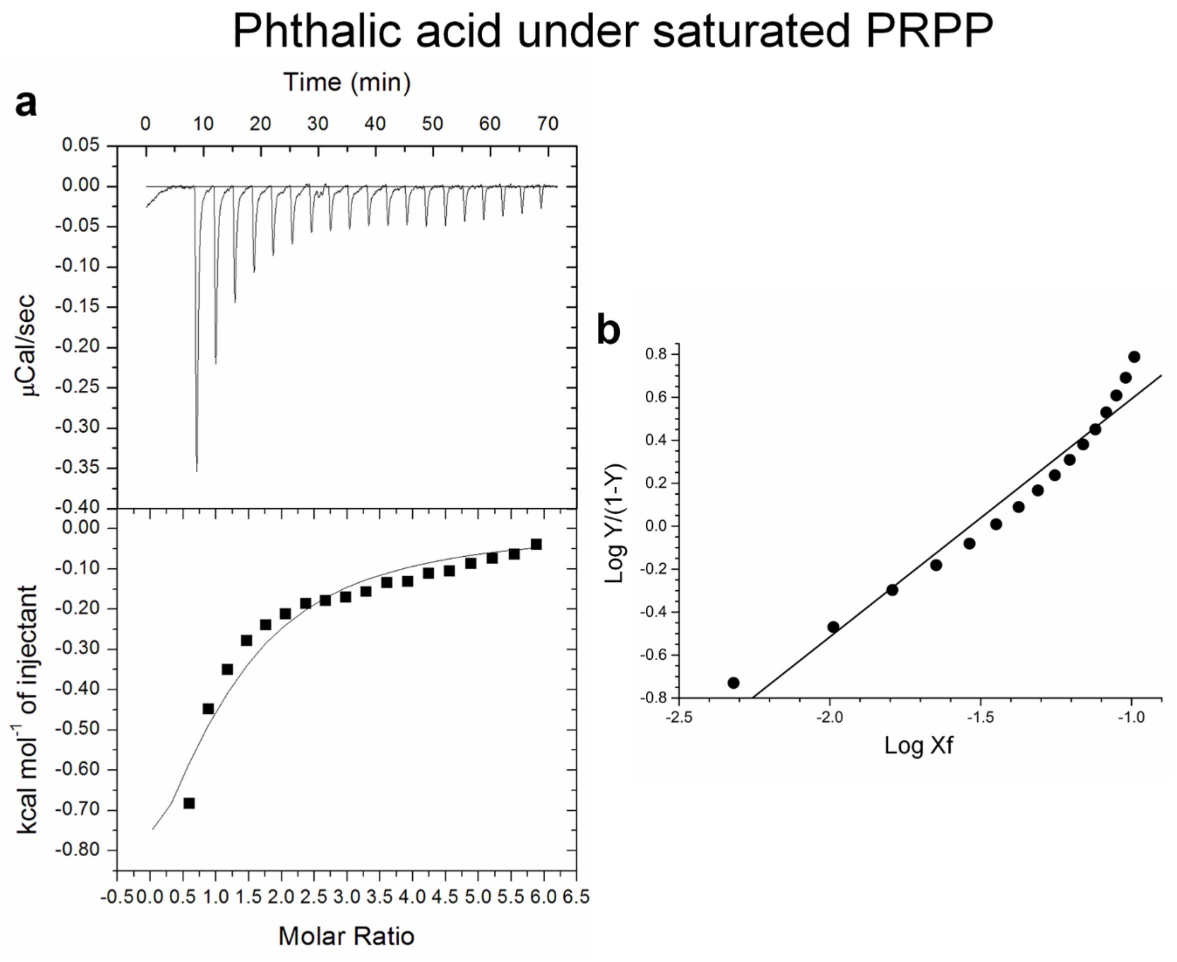

Supplementary Figure S11. (a) ITC profile for the binding of phthalic acid (800 μM) to *Hs*QPRT (30 μM) under saturated PRPP (300 μM) with N (stoichiometry) = 1.0, K_d = ~33 μM, ΔH = ~-1.8 kcal/mol and ΔS = ~14.2 cal/mol. (b) Hill plot derived from the ITC data.

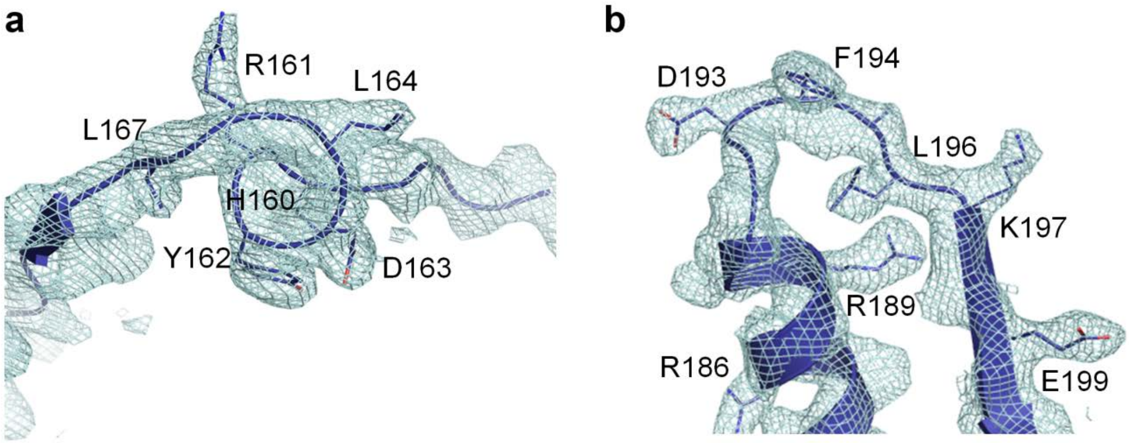

Supplementary Figure S12. The electron density corresponds to loop L (a) and loop O (b) of MolA of *Hs*QPRT-open contoured at 1.0 σ.

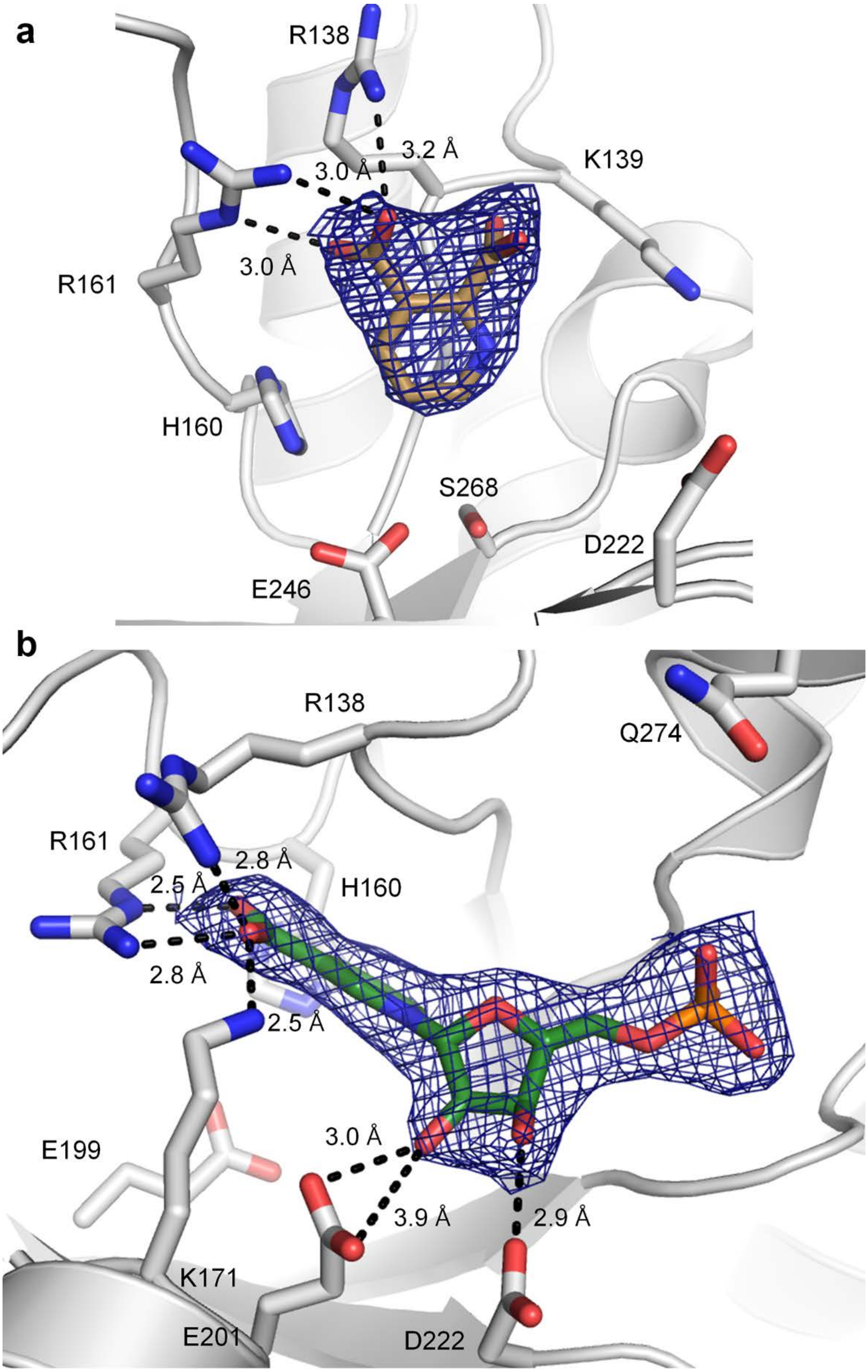

Supplementary Figure S13. Simulated-annealing composite omit map of QA (a) and NAMN (b) molecules. The electron density was contoured at 1.0 σ. Hydrogen bonds are indicated as dashed lines.
